# Comparison of computed tomography perfusion and magnetic resonance dynamic susceptibility contrast perfusion-weighted imaging in canine brain

**DOI:** 10.3389/fvets.2024.1298215

**Published:** 2024-03-11

**Authors:** Sangmin Lee, Soomin Park, Sungwha Hong, Soyeon Kim, Junghee Yoon, Jihye Choi

**Affiliations:** Department of Veterinary Medical Imaging, College of Veterinary Medicine, Seoul National University, Seoul, Republic of Korea

**Keywords:** cerebral, CTP, dog, DSC, perfusion weighted imaging

## Abstract

Brain perfusion allows for the evaluation of cerebral hemodynamics, particularly in brain infarcts and tumors. Computed tomography (CT) perfusion (CTP) provides reliable data; however, it has a limited scan field of view and radiation exposure. Magnetic resonance (MR) perfusion provides detailed imaging of small structures and a wide scan field of view. However, no study has compared CTP and MR perfusion and assessed the correlation between the perfusion parameters measured using CTP and MR perfusion. The aim of the present study was to assess the correlation and agreement of the cerebral perfusion derived from dynamic susceptibility contrast (DSC)-MRI and CTP in dogs. In this crossover design study, the cerebral blood volume (CBV), cerebral blood flow (CBF), mean transit time, and time to peak were measured in the temporal cerebral cortex, caudate nucleus, thalamus, piriform lobe, and hippocampus using CTP and DSC-MRI in six healthy beagle dogs and a dog with a pituitary tumor. On the color map of healthy beagles, blood vessels and the perivascular brain parenchyma appeared as red-green, indicating high perfusion, and the areas distant from the vessels appeared as green-blue, indicating low perfusion levels in CTP and DSC-MRI. CTP parameters were highest in the piriform lobe (CBF = 121.11 ± 12.78 mL/100 g/min and CBV = 8.70 ± 2.04 mL/100 g) and lowest in the thalamus (CBF = 63.75 ± 25.24 mL/100 g/min and CBV = 4.02 ± 0.55 mL/100 g). DSC-MRI parameters were also highest in the piriform lobe (CBF = 102.31 ± 14.73 mL/100 g/min and CBV = 3.17 ± 1.23 mL/100 g) and lowest in the thalamus (CBF = 37.73 ± 25.11 mL/100 g/min and CBV = 0.81 ± 0.44 mL/100 g) although there was no statistical correlation in the quantitative perfusion parameters between CTP and DSC-MRI. In a dog with a pituitary tumor, the color map of the tumor appeared as a red scale, indicating high perfusion and higher CBF and CBV on CTP (149 mL/100 g and 20 mL/100 g/min) and on DSC-MRI (116.3 mL/100 g and 15.32 mL/100 g/min) compared to those measured in healthy dogs. These findings indicate that DSC-MRI and CTP maps exhibit comparability and interchangeability in the assessment of canine brain perfusion.

## 1 Introduction

Magnetic resonance imaging (MRI) is mainly used to diagnose intracranial disorders in dogs because of its higher resolution of the soft tissues compared with computed tomography (CT) (1). The administration of intravenous contrast agents can improve the conspicuity of intracranial lesions, visualization of blood vessels, and even provide information about perfusion (2). Perfusion refers to the flow of oxygenated blood from the arteries to the tissues through capillaries and perfusion is related to the oxygen and nutrient supply to the tissues (1). In human patients with brain disorders including strokes, cerebrovascular accidents, tumors, encephalitis, and degenerative diseases, the clinical evaluation of brain perfusion is an essential tool to diagnose and make a plan for the disease and predict the prognosis (2, 3). In particular, the clinical role of perfusion imaging is well-known in cerebrovascular accidents for distinguishing between an infarction core, characterized by irreversibly reduced blood supply, and a penumbra, which comprises viable tissue surrounding the infarction core (4).

For the perfusion analysis, CT perfusion (CTP) is considered the gold standard for identifying ischemia because of its high sensitivity and specificity in both animals and humans. Moreover, its shorter scanning time can reduce the duration of anesthesia and total examination compared to MR perfusion (4). However, due to concerns about radiation exposure and limited scan field of view (FOV) for CTP, the alternative use of MR perfusion has been tried to assess brain perfusion. In addition, newly developed perfusion weight sequences for MRI make MR perfusion the potential tool for cerebral perfusion analysis with its intrinsic high spatial resolution and wide scan FOV (5).

In CTP, after a single injection of an iodine contrast medium, the change in pixel enhancement of the cerebral artery and vein can be measured in Hounsfield unit (HU) by placing regions of interest (ROIs) over the middle cerebral artery, dorsal sagittal sinus, and brain tissue and generating the time-density curve (TDC) (6, 7). Then, a perfusion map can be obtained from the TDC, and the quantitative perfusion parameters including cerebral blood volume (CBV), cerebral blood flow (CBF), time to peak of tissue enhancement (TTP), and mean transit time (MTT) can be measured (8, 9).

MR perfusion can be performed using three techniques including DSC, dynamic contrast-enhanced (DCE), and arterial spin labeling (ASL) and perfusion parameters such as CBV, CBF, TTP, MTT, negative enhancement integral (NEI), and k-trans can be derived. For MR perfusion, ASL uses an endogenous contrast agent meanwhile DSC and DCE-MRI use the exogenous gadolinium-based contrast agents for perfusion imaging (10). DSC-MRI is most commonly used for cerebral perfusion analysis with dedicated software (11, 12). DSC-MRI captures the initial passage of the intravenous injection of the contrast agent in the brain using a T2*-weighted (T2*W) MR sequence and generates a signal intensity-time curve, referred to as an arterial input function (AIF) (13, 14). The susceptibility made by the contrast agent reduces the signal intensity in the AIF and provides the color map and perfusion parameters like CBV, CBF, TTP, and MTT (10).

Although DSC-MRI and CTP have differences in the process of acquisition and computational models, both techniques use monitoring the delivery of contrast to tissues after the bolus injection of the contrast agent and the deconvolution method to analyze cerebral perfusion (15, 16). Therefore, DSC-MRI could be used interchangeably or as an alternative to CTP when radiation exposure is the main concern or the whole brain could be included in FOV for brain perfusion. However, the comparison between CTP and MR perfusion parameters has been assessed in only two human studies on brain tumors (5, 17). One study described the concordance in CBV measurement between DSC-MRI and CTP in human patients with high-grade glioma (5). The others have estimated the correlations in permeability measurements between DCE-MRI and CTP in patients with brain metastasis and glioblastoma (17). However, to the authors’ knowledge, there is no study to compare brain perfusion performed by CTP and DSC-MRI in veterinary medicine. Therefore, in this study, brain perfusion was performed using CTP and DSC-MRI in clinically normal dogs and the color map and perfusion parameters obtained by the two techniques were compared with each other. We hypothesized that the perfusion parameters derived from DSC-MRI and CTP would be comparable and interchangeable.

The purpose of this study was to evaluate the feasibility of DSC-MRI for brain perfusion and assess the correlation of MR perfusion parameters with CTP parameters in clinically normal beagle dogs.

## 2 Materials and methods

This study was approved by the Institutional Animal Care and Use Committee at Seoul National University, and the dogs were cared for according to the Guidelines for Animal Experiments of Seoul National University (SNU IACUC-SNU-230801-5).

### 2.1 Study design

- Study 1. CTP and DSC-MRI in normal beagles

In this prospective, experimental study, six (four intact male and two intact female) purpose-bred beagles were used. The median age was 3.5 years (2–5 years) and the median weight was 13 kg (9.9–15.5 kg). All dogs were in good health based on blood pressure, physical examination, urinalysis including urine dipstick and urine specific gravity, complete blood count, serum biochemistry, abdominal ultrasonography, echocardiography, and thoracic and abdominal radiography. All dogs had no history of neurologic signs or intracranial disorders. Dogs were kept in an air-conditioned room in individual cages and fed commercial food and water *ad libitum*.

- Study 2. CTP and DSC-MRI in a dog with a pituitary tumor

A 12-year-old Spayed female Pomeranian suspected of a pituitary tumor presented to Seoul National University Veterinary Medical Teaching Hospital for confirmed diagnosis and radiation therapy. The dog showed neurological symptoms such as left-sided circling, mild ataxia, and stupor for 6 months. In the dog, a pituitary gland tumor was diagnosed at a local animal hospital based on MR images revealing a mass measuring 16.6 × 13.8 × 17.6 mm in the sellar region. On MR images, the mass was observed on T2-weighted (T2W) heterogenous hyperintense, T1-weighted (T1W) iso-hypointense, and fluid-attenuated inversion recovery (FLAIR) hyperintense and had well-defined margin and intratumoral hemorrhage. The mass displaced the cranial part of the midbrain and an upward shift of the hypothalamus. Based on the mass size and location, a macroadenoma originating at the pituitary gland was considered and an adenoma or adenocarcinoma was suspected. CT and MRI scans, including perfusion imaging, were performed for radiotherapy planning. In the dog, CTP and DSC-MRI were conducted using the same method as that employed in Study 1.

### 2.2 Anesthesia and schedule

CT and MRI of the brain were performed under general anesthesia with at least 5-day intervals in random order. After fasting the dog for at least 8 h, a 24-gauge catheter was aseptically placed into a cephalic vein. Anesthesia was induced with intravenous injection of 10–15 μg/kg of medetomidine hydrochloride (Domitor, Zoetis, Finland) and 2 mg/kg of alfaxalone (Alfaxan, Jurox Pty Ltd., Australia). Following endotracheal intubation, anesthesia was maintained with isoflurane (2–4%; Ifran, Hana Pharm, South Korea) delivered in 100% oxygen (1–2 L/min). During the induction and maintenance of anesthesia, heart rate and oxygen saturation were continuously monitored with a respiratory sensor, electrocardiography (ECG) tracing, and pulse oximetry. Following the MRI and CT scans, the overall condition of each dog and any anesthesia-related side effects, including vomiting, depression, and anorexia, were monitored for a duration of 5 days.

### 2.3 Conventional MRI and DSC-MRI

After placing the dog in sternal recumbency, an MRI was performed using a 1.5 T-MRI scanner (GE Signa 1.5 T, GE Healthcare, Illinois, US) with an 8-channel knee coil using the following sequences: dorsal plane of T2W, sagittal planes of T2W, sagittal T1W, and transverse planes of T1W, T2W, FLAIR, and post-contrast T1W. The scan FOV covered from the nostril to the occipital bone.

DSC-MRI of the brain was performed using a T2*W echo planar image sequence on the transverse plane perpendicular to the skull base. For each slice, 40 dynamics were acquired with a 1.6-s interval between each slice. Each dynamic lasted for 1.5–2 s depending on the number of slices. Continuous transverse slices were taken throughout the brain for each measurement. The image acquisition parameters were as follows: flip angle = 60°, slice thickness = 4 mm, FOV = 140 cm, number of excitations = 1, matrix size = 96 × 84, and repetition time = 1,500 ms. A paramagnetic gadolinium-based contrast agent (Dotarem, Guerbet, France) was administered through a cephalic vein catheter using an automatic power injector after five dynamic images. Consequently, the first five images represented the baseline, and the images from six dynamics provided the flow dynamics of the contrast agent. All dogs received a 0.2 mL/kg contrast bolus at 2 mL/s followed by a 10 mL saline flush at 2 mL/s. Following the acquisition of perfusion data, post-contrast transverse T1W images were obtained for all dogs.

### 2.4 Conventional CT and CTP examinations

After positioning the dog in a sternal recumbent posture, with the head at the isocenter of the gantry, CT images were acquired using a 160-multislice CT scanner (Aquilion Lightning 160 MODEL TSX-036A, Canon Medical System, Japan). A pre-contrast CT scan was conducted from the nose to the first cervical vertebrae using the following parameters: tube voltage = 100 kVp, tube current = 400 mAs, rotation time = 0.75 s, pitch = 0.8, and slice thickness = 0.5 mm. Based on the pre-contrast image, a range from the parietal lobe to the cerebellum, including the hippocampus and the occipital lobe, was chosen for brain CTP. Then, CTP scanning was performed at 2.7-s intervals for a duration of 57.45 s concurrently with the initiation of injection of 1 mL/kg contrast agent and 350 mg iodine/mL of iohexol (Omnipaque, GE Healthcare,), via a 22-gauge intravenous catheter utilizing a power injector at a rate of 2 mL/s.

### 2.5 Post-processing and analysis of CTP and DSC-MRI

**Figure 1:**
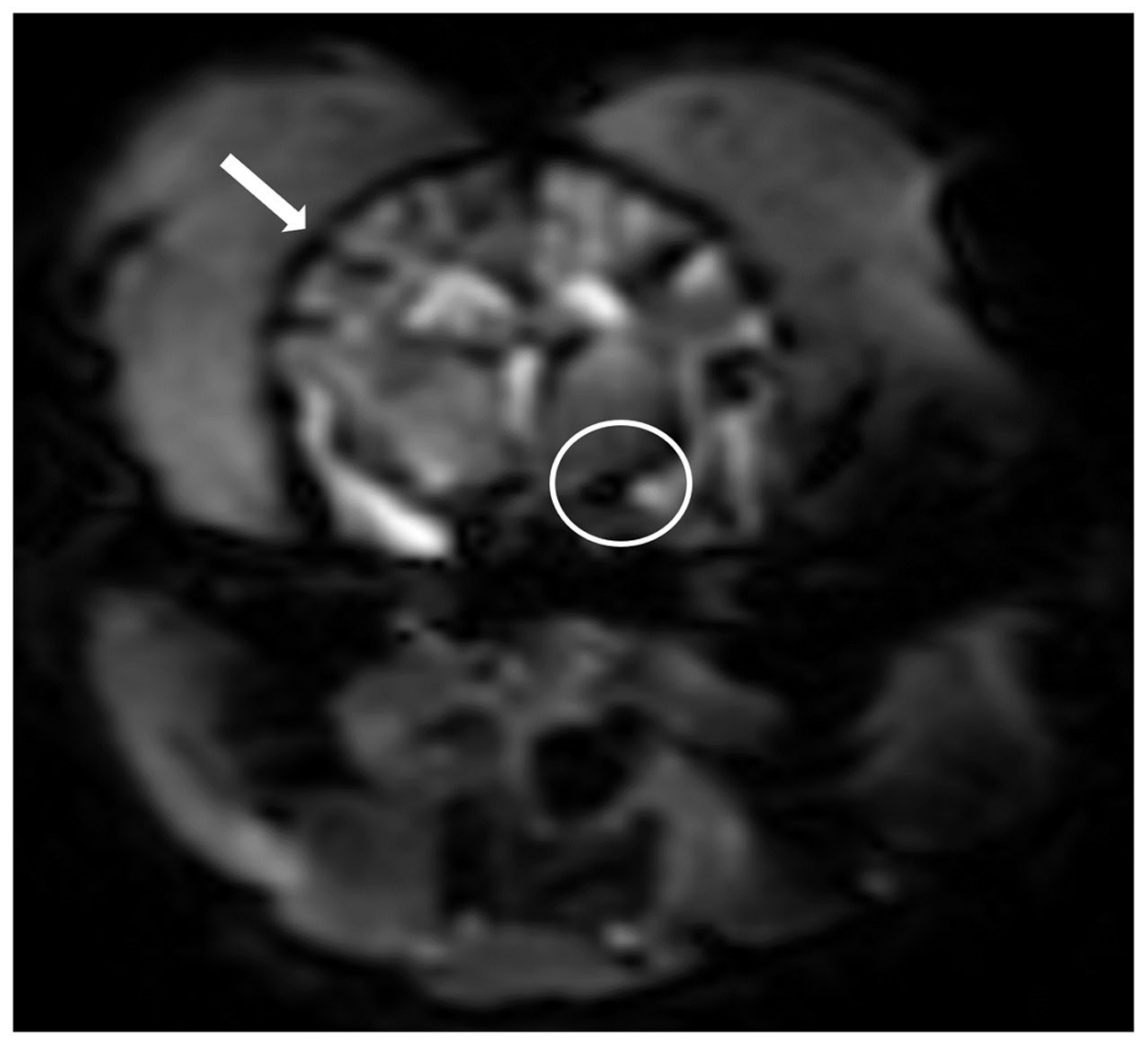
T2*-weighted imaging sequence after the administration of a gadolinium-based contrast agent at the middle cerebral artery level. Note hypointense cortical arteries at the outer edges of the cerebrum (arrow) and the middle cerebral artery (circle) showing peak contrast susceptibility. Perfusion analysis can be performed after placement of a region of interest over the middle cerebral artery and obtained an arterial input function graphs.

**Figure 2:**
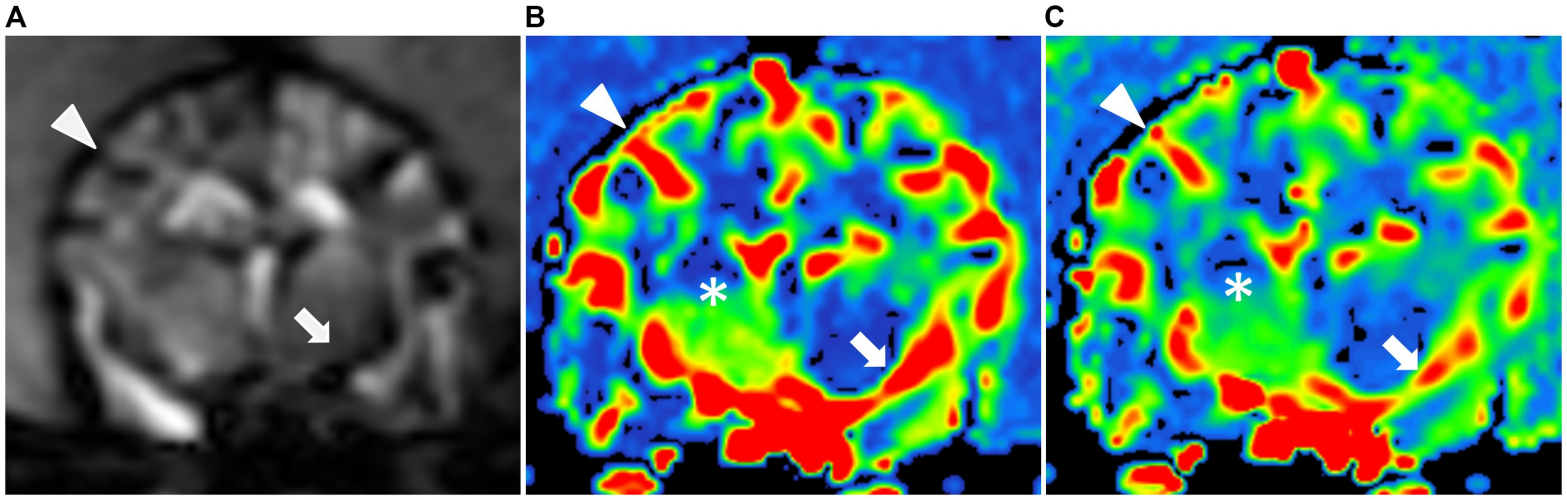
Dynamic susceptibility contrasts MR perfusion in the normal canine brain. **(A)** Dynamic susceptibility contrast MR T2*-weighted image at the middle cerebral artery level: Note the hypointensity of the cortical arteries (black arrowhead) and the middle cerebral artery (black arrow) during the first passage of the gadolinium contrast bolus in the arterial phase. Color maps of the cerebral blood flow **(B)** and cerebral blood volume **(C)** at the middle cerebral artery level: These maps use a color scale ranging from blue (indicating low perfusion) to red (indicating high perfusion). Note the red-colored middle cerebral arteries and the red-to-green cortical arteries (white arrow and arrowhead) as well as the blue-to-green cerebral parenchyma (white asterisks).

Post-processing of DSC-MRI images was conducted at the workstation using the installed perfusion analysis software (AW server readyview, GE Healthcare, Milwaukee, Wisconsin) by one of the authors (SL). First, an ROI was positioned over the middle cerebral artery. Then, multiple AIF graphs were generated and showed the concentration of the contrast agent in an artery over time (Figure 1). These curves were then averaged by the software to create a representative averaged AIF to generate cerebral perfusion maps. Parametric maps of CBF and CBV were generated using the standard truncated singular value decomposition algorithm for deconvolution, along with an automated method to obtain the AIF. The software produced color perfusion maps consisting of blue, green, and red for CBV and CBF maps, and red indicated high perfusion, dark blue indicated very low perfusion, and green indicated intermediate perfusion (Figure 2). CBV was represented by the integrated area under the AIF curve, relative MTT was reflected by the width of the curve, and CBF was determined as the ratio of CBV to MTT.

**Figure 3:**
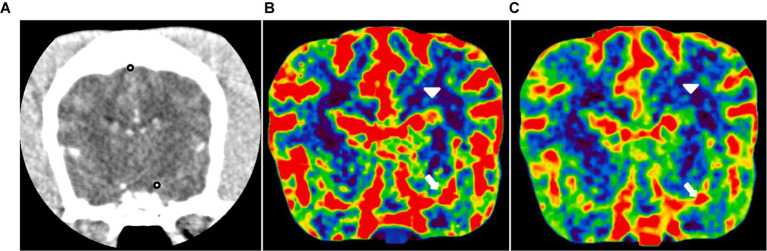
CT perfusion in the normal canine brain. **(A)** CT image at the level of the middle cerebral artery and dorsal sagittal sinus (black dot). Cerebral blood flow **(B)** and cerebral blood volume **(C)** maps at the level of the middle cerebral artery. These maps utilize a color scale ranging from blue (indicating low perfusion) to red (indicating high perfusion). Note the red-colored middle cerebral arteries (white arrow) and the blue-to-green cerebral parenchyma (white arrowhead).

CTP maps were generated using an installed delay-insensitive deconvolution software (VITREA^®^, Canon Medical Systems). For the TDC, the middle cerebral artery and dorsal sagittal sinus were manually selected as ROIs (Figure 3). Arterial and venous TDCs were obtained by placing two ROIs with 2 × 2 voxels in-slice for each. Then, five perfusion parameters including CBV, CBF, TTP, and MTT were measured from the TDC.

In both CTP and DSC-MRI, tissue perfusion was evaluated from five evaluating sites such as the temporal cerebral cortex, caudate nucleus, thalamus, hippocampus, and piriform lobe. These evaluating sites were manually delineated on one side and then mirrored to estimate the symmetrically opposite side at the same level in accordance with the previous study (18). All perfusion data were independently assessed by two observers (SL and SP), each with 2 years of radiology experience, in a blinded manner.

### 2.6 Statistical analyses

Statistical analyses were performed by a statistician (JP) using commercially available software (SPSS Statistics, Version 27 for Windows, IBM Corp., Chicago, IL, United States). Spearman’s rho test was used to analyze the concordance in perfusion parameters between DSC-MRI and CTP. To examine whether there were any patterns between DSC-MRI and CTP parameters, a run test was conducted. All data are presented as the mean ± standard deviation. Statistical significance was set at *p* < 0.05.

## 3 Results

### 3.1 Study 1: CTP and DSC-MRI in normal beagles

CTP and DSC-MRI were performed successfully without any complications and the color maps and quantitative perfusion parameters were obtained in all dogs.

**Figure 4:**
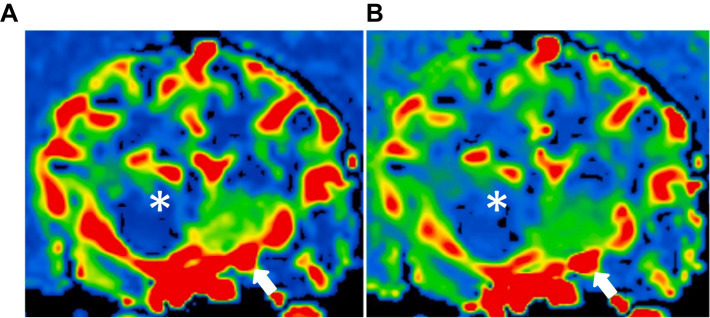
Dynamic susceptibility contrasts MR perfusion in the normal canine brain. **(A)** and **(B)** are different dogs, demonstrating similarity in the color maps between the right and left hemispheres. These maps utilize a color scale ranging from blue (indicating low perfusion) to red (indicating high perfusion). Note the red-colored piriform lobe (white asterisk) and the blue-to-green thalamus (asterisk).

DSC-MRI provided a similar color map of the canine brain with CTP. In the color map obtained by the two perfusion techniques, the piriform lobe appeared to be the most prominently red, indicating high perfusion. Conversely, the thalamus exhibited green perfusion, signifying the lowest perfusion among the five evaluating sites. In contrast, the caudate nucleus, temporal cerebral cortex, and hippocampus displayed a similar degree of red-green coloration. In addition, there was no significant difference in mean values of the perfusion parameters and color map measured from each evaluating site between the right and left hemispheres (Figure 4).

In a quantitative assessment, the mean values of CTP parameters (Table 1) and DSC-MRI (Table 2) of the five evaluating sites were presented. There was no significant difference in mean values of the CTP parameters measured from each evaluating site between the right and left hemispheres. The mean value of CBV in each evaluating site was listed from high to low in the following order: piriform lobe, caudate nucleus, temporal cerebral cortex, hippocampus, and thalamus in both DSC-MRI and CTP. The mean value of CBF was listed from high to low in the same order in both DSC-MRI and CTP. These findings were consistent with the color map results. Consequently, it was confirmed that perfusion was highest in the piriform lobe and lowest in the thalamus. The perfusion parameters measured by DSC-MRI did not show a significant correlation with those of CTP regardless of the evaluating sites (Tables 3, 4).

**Table 1:** CT perfusion parameters in five regions of the brain in six healthy adult beagle dogs.

Evaluating sites	CBF (mL/100 g/min)	CBV (mL/100 g)	MTT (s)	TTP (s)
Caudate nucleus	107.23 ± 17.47 (77–145.70)	6.59 ± 1.74 (3.40–9.40)	3.57 ± 1.11 (1.70–4.90)	17.13 ± 4.02 (12.30–23.50)
Thalamus	63.75 ± 25.24 (41.90–114.10)	4.02 ± 0.55 (3.00–4.70)	4.68 ± 1.12 (2.30–5.50)	17.98 ± 3.96 (13.50–23.90)
Piriform lobe	121.11 ± 12.78 (93.90–140.20)	8.70 ± 2.04 (5.50–11.30)	3.93 ± 0.72 (3.93–0.72)	16.92 ± 3.52 (12.90–21.80)
Hippocampus	92.82 ± 26.21 (65.10–146.70)	5.73 ± 0.74 (4.70–7.00)	4.29 ± 1.13 (2.00–5.70)	17.86 ± 4.77 (13.00–26.80)
Temporal cerebral cortex	103.80 ± 21.98 (103.80–21.98)	6.55 ± 1.01 (4.30–7.80)	4.13 ± 1.28 (2.20–6.90)	17.09 ± 4.23 (11.80–23.60)

CBF, cerebral blood flow; CBV: cerebral blood volume; MTT, mean transit time; TTP, time to peak. Data are presented as mean ± standard deviation (ranges).

**Table 2:** Dynamic susceptibility contrast-MR perfusion parameters in five regions of the brain in six healthy adult beagle dogs.

Evaluating sites	CBF (mL/100 g/min)	CBV (mL/100 g)	MTT (s)	TTP (s)
Caudate nucleus	76.71 ± 24.53 (35.16–131.00)	3.10 ± 1.43 (1.08–4.90)	2.03 ± 0.64 (1.39–3.47)	36.39 ± 3.00 (32.45–41.58)
Thalamus	37.73 ± 25.11 (11.83–87.62)	0.81 ± 0.44 (0.15–1.68)	1.60 ± 0.42 (0.99–2.27)	33.83 ± 5.02 (19.99–39.39)
Piriform lobe	102.31 ± 14.73 (83.09–132.30)	3.17 ± 1.23 (3.17–1.23)	1.79 ± 0.83 (1.09–3.30)	34.67 ± 3.14 (29.87–39.49)
Hippocampus	73.10 ± 34.69 (24.11–134.50)	1.79 ± 0.72 (0.48–3.13)	1.55 ± 0.34 (1.02–2.13)	33.97 ± 6.64 (18.72–41.06)
Temporal cerebral cortex	75.11 ± 30.89 (32.93–122.20)	3.01 ± 1.88 (0.36–6.03)	2.14 ± 1.08 (0.95–4.35)	34.14 ± 4.93 (26.96–42.87)

CBF, cerebral blood flow; CBV: cerebral blood volume; MTT, mean transit time; TTP, time to peak. Data are presented as mean ± standard deviation (ranges).

**Table 3:** The correlation coefficient of cerebral blood flow measured by CT perfusion and dynamic susceptibility contrast MR perfusion in five regions of the brain.

Regions of interest	Correlation coefficient	*p* value
Caudate Nucleus	−0.070	0.829
Hippocampus	0.469	0.124
Piriform lobe	−0.196	0.542
Temporal cortex	0.336	0.286
Thalamus	0.091	0.779

**Table 4:** The correlation coefficient of cerebral blood volume measured by CT perfusion and dynamic susceptibility contrast MR perfusion in five regions of the brain.

Regions of interest	Correlation coefficient	*p* value
Caudate Nucleus	−0.130	0.688
Hippocampus	−0.264	0.433
Piriform lobe	0.119	0.713
Temporal cortex	0.021	0.948
Thalamus	−0.268	0.400

### 3.2 Study 2: CTP and DSC-MRI in a patient with a pituitary tumor

**Figure 5:**
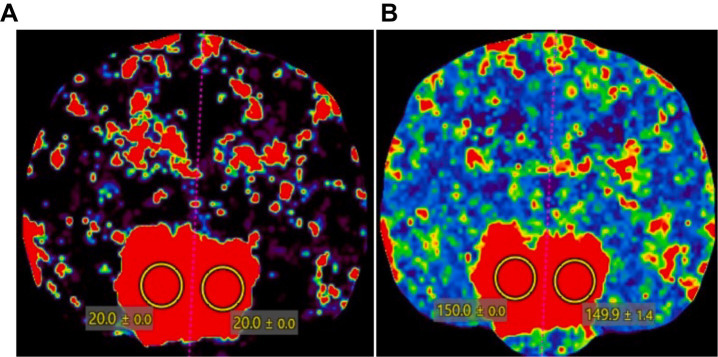
CT perfusion color map of a dog with a pituitary tumor. A pituitary mass showed a red scale, indicating high perfusion in **(A)** cerebral blood volume and **(B)** cerebral blood flow. Two regions of interest were drawn over the pituitary gland to measure the CT perfusion parameters.

On CTP and DSC-MRI of the pituitary tumor in a dog, the color map of the tumor appeared as a red scale, indicating high perfusion (Figure 5). However, the regions outside the tumor area in the patient, especially the five evaluation sites (piriform lobe, caudate nucleus, temporal cerebral cortex, hippocampus, and thalamus), displayed color maps and parameter values similar to those of normal dogs. In the quantitative evaluation of the pituitary tumor in a dog, CBF and CBV measured using CTP were 149 mL/100 g and 20 mL/100 g/min, respectively, which were higher than those measured in normal dogs in Study 1. CBF and CBV measured using DSC-MRI in the patient were 116.3 mL/100 g and 15.32 mL/100 g/min, respectively, which were also higher than those measured in healthy dogs in Study 1.

## 4 Discussion

This study evaluated brain perfusion using CTP and DSC-MRI in clinically normal dogs and compared the color map and perfusion parameters with each other to assess the feasibility of MR perfusion for the brain and the correlation of the perfusion parameters measured by both modalities in clinically normal beagle dogs. There was no statistically significant variation in the quantitative comparison between the two cerebral hemispheres, and no significant difference was noted in the qualitative evaluation either. We presented normal values of CBV, CBF, TTP, and MTT using CTP and DSC-MRI in beagle dogs.

DSC-MRI and CTP showed similar perfusion color maps in the evaluating sites of the brain parenchyma. These maps utilize a color scale ranging from blue to red, where red represents high perfusion, and blue indicates low perfusion. In the color map, the piriform lobe appeared as the most prominently red, indicating high perfusion. Conversely, the thalamus exhibited green perfusion, signifying the lowest perfusion among the five evaluating sites. The caudate nucleus, temporal cerebral cortex, and hippocampus displayed a similar degree of red-green colors.

In addition, a quantitative assessment of CBF and CBV via DSC-MRI and CTP revealed consistent perfusion measurements in the following order: piriform lobe, caudate nucleus, temporal cerebral cortex, hippocampus, and thalamus. These findings were consistent with the color map results. Consequently, it was confirmed that perfusion was highest in the piriform lobe and lowest in the thalamus. Although the previous study’s results and the quantitative evaluation values did not match exactly, when considering the order of highest to lowest mean values, they exhibited a similar trend (18).

In our study, there was no significant difference in mean values of the perfusion parameters measured from each evaluating site between the right and left hemispheres. The presence of differences in the perfusion between the left and right hemispheres is controversial. A previous human study reported significantly higher brain perfusion parameters in the left cerebral hemisphere compared to the right, which is possibly volume differences (19). Similar observations have been made in dogs using SPECT (20). However, other studies using SPECT and MR perfusion did not find significant differences in perfusion between the left and right cerebral hemispheres in dogs (21).

For the quantitative perfusion parameters, when separately comparing CTP and DSC-MRI values within the normal group, significant differences were observed for the designated ROI in each case. In a prior human study, DSC-MRI and CTP yielded similar results when comparing mean CBV and mean CBF values in high-grade and low-grade gliomas, as all these parameters were higher in high-grade gliomas compared to low-grade gliomas (5). However, this study is discordant with the results obtained from other previous human studies. Possible explanations for these discrepancies between CTP and DSC-MRI include the following. First, the subjects of evaluation are different. This study assessed five ROIs of the normal brain in six healthy beagles, not tumors in patients. Since ROI selection was done manually, there may have been slight variations in the selected areas for each individual. Therefore, when the areas outside the ROI were included in addition to the perfusion area, it could have introduced some differences in perfusion values, which might explain the disparities observed when comparing CTP and DSC-MRI (22). Second, the different deliveries of the contrast agents may influence the perfusion measurements. In this study, MRI utilized a dual injector, whereas CT utilized a single injector for delivery of the contrast agents. There might have been differences between the two modalities regarding the administration of saline. Both experiments administered the contrast agent at the same injection rate (2 mL/s), but there was a difference in the degree of washout through saline. While the contrast agent injection went well, there were differences in the perfusion levels. This indicates that variations in injection parameters could also have contributed to the differences in results (23–25). Third, vendors’ software differentiation may affect the quantitative perfusion results (26, 27).

However, when this study was applied to a patient with a pituitary tumor, the results were similar to the human study, with CBV and CBF values showing a close resemblance. This is likely attributed to the evaluation of a single region. By referring to a single region, it means that the same area, corresponding to the sellar region in patients with tumors, was evaluated consistently in the beagles as well. In quantitative evaluations, there was no significant correlation. However, there was a similarity observed in color map evaluations, especially in regions with high and low perfusions. Comparing CTP and DSC-MRI for the control group of six beagles revealed consistent values, and there was a similar tendency in regions with high and low perfusions in both CTP and DSC-MRI. The experiment comparing patients to the control group revealed that the perfusion values in patients were higher than those in the control group. This demonstrates elevated perfusion in tumors, indicating a higher perfusion parameter value, thereby confirming the presence of increased blood flow in tumor areas (3, 28, 29).

In this study, five evaluating sites including the temporal cerebral cortex, caudate nucleus, thalamus, hippocampus, and piriform were selected for analyzing brain perfusion because they were visible on DSC-MRI and CTP images, have different functions, and are important in various brain diseases. These regions are susceptible to specific disorders based on their characteristics, especially the caudate nucleus, which can be compressed due to hydrocephalus because of its proximity to the lateral ventricles, and the thalamus, which is prone to seizure disorders. Furthermore, seizures can also affect the piriform lobe and temporal lobe (30–32). Additionally, the hippocampus is known to be particularly susceptible to the impact of epilepsy (33, 34). Therefore, perfusion assessment of these brain regions can be instrumental in confirming the presence of related conditions.

This study has several limitations. First, this study was performed only in a small number of normal beagle dogs with the same body size and skull shape. Second, we presented only one case with a pituitary tumor assessed by CTP and DSC-MRI. In clinical practice, it is not easy to perform both CTP and DSC-MRI in patients with brain tumors. However, the perfusion changes in brain tumors should be evaluated using CTP and MR perfusion in large populations of animal patients with brain tumors. Third, CTP and DSC-MRI were performed in all dogs under anesthesia. The anesthesia would affect the TDC graphs and perfusion parameters because isoflurane inhalation used in our study is known to have an effect on cerebral vasodilation, causing an increase in CBF, cerebral autoregulation and metabolism, and even functional activity of the brain (35–37). Some studies in monkeys have demonstrated a significant increase in CBF in the cerebrum, medulla, and cerebellum when a high dose (2.0%) of isoflurane is given (38). However, in our study, the comparison of cerebral perfusion measured by CTP and DSC-MR is the main topic, and the heart rate, blood pressure, and respiratory rates were not different during CTP and DSC-MRI in dogs. Therefore, the anesthetic effect on the perfusion parameters would be consistent in each dog regardless of the perfusion techniques.

This study represents the initial exploration of the distinctions and commonalities in brain perfusion parameters across various canine brain regions as identified through DSC-MRI and CTP. To the best of the authors’ knowledge, this study is the first in veterinary medicine to compare CTP and DSC-MRI. Collectively, our findings suggest that DSC-MRI and CTP-derived CBF and CBV maps exhibit comparability and interchangeability in the assessment of brain diseases. This finding is of clinical significance since it is often difficult to perform both CTP and DSC-MRI simultaneously in patients in clinical practice. Consequently, when DSC-MRI is unavailable, CTP could serve as a viable alternative to quantify brain neoangiogenesis reliably despite concerns related to radiation exposure. Additionally, CTP is a suitable option when DSC-MRI cannot be performed due to patient contraindications or intolerance. Nevertheless, in specific cases, DSC-MRI may be preferred due to its superior soft tissue contrast and the absence of ionizing radiation for brain perfusion. In summary, the choice between CTP and DSC-MRI can be made based on the specific clinical scenario and requirements. Future studies should aim to gather a more extensive dataset using CTP or DSC-MRI in dogs of varying sizes and breeds to enhance the overall utility of these imaging modalities.

## Data availability statement

The original contributions presented in the study are included in the article/supplementary material, further inquiries can be directed to the corresponding author.

## Ethics statement

The animal studies were approved by the Institutional Animal Care and Use Committee at Seoul National University, and the dogs were cared for according to the Guidelines for Animal Experiments of Seoul National University (SNU IACUC-SNU-230801-5). The studies were conducted in accordance with the local legislation and institutional requirements. Written informed consent was obtained from the owners for the participation of their animals in this study.

## Author contributions

SL: Data curation, Formal analysis, Investigation, Methodology, Visualization, Writing – original draft. SP: Data curation, Formal analysis, Writing – review & editing. SH: Data curation, Methodology, Writing – review & editing. SK: Data curation, Methodology, Writing – review & editing. JY: Writing – review & editing. JC: Conceptualization, Funding acquisition, Project administration, Supervision, Validation, Writing – review & editing.
